# CDK4, pRB and E2F1: connected to insulin

**DOI:** 10.1186/1747-1028-5-6

**Published:** 2010-02-05

**Authors:** Lluis Fajas, Emilie Blanchet, Jean-Sébastien Annicotte

**Affiliations:** 1 IRCM, Institut de Recherche en Cancérologie de Montpellier, Montpellier, F-34298, France; 2 INSERM, U896, Montpellier, F-34298, France; 3 CRLC Val d'Aurelle Paul Lamarque, Montpellier, F-34298, France; 4 Univ Montpellier1, Montpellier, F-34298, France

## Abstract

Pancreatic β-cells are metabolic sensors involved in the control of glucose homeostasis. This particular cell type controls insulin secretion through a fine-tuned process, which dregulation have important pathological consequences, such as observed during type 2 diabetes. We recently implicated E2F1 in the control of glucose homeostasis. First we showed that *E2f1*-/- mice have decreased pancreatic size, as the result of impaired postnatal pancreatic growth. We observed in this study that E2F1 was highly expressed in non-proliferating pancreatic β-cells, suggesting that E2F1, besides the control of β-cell number could have a role in pancreatic β-cell function. We demonstrate in our recent study, both *in vitro *and *in vivo *that E2F1 directly regulates the expression of *Kir6.2*, a key component of the K_ATP _channel involved in the regulation of glucose-induced insulin secretion in pancreatic β-cells. Expression of *Kir6.2 *is lost in pancreas of *E2f1*-/- mice, resulting in insulin secretion defects in these mice. Furthermore, we demonstrated by *in tissue *chromatin immunoprecipitation analysis that regulation of *Kir6.2 *expression by E2F1 follows the same regulatory pathway that the classical E2F1 target genes, implicating the participation of CDK4 and retinoblastoma protein. Moreover, in this context, E2F1 transcriptional activity is regulated by glucose and insulin through the CDK4-dependent inactivation of the pRB protein. In summary we provide evidence that the CDK4-pRB-E2F1 regulatory pathway is involved in glucose homeostasis. In our recent study we decipher a new function for these factors in the control of insulin secretion and open up new avenues for the treatment of metabolic diseases, in particular type 2 diabetes.

## Introduction

The synthesis of insulin occurs in a specific compartment of the islet of Langerhans, the pancreatic β-cells. In normal conditions, transient hyperglycemia requires increased insulin secretion, which could be obtained through several adaptations of the β-cell, *i.e*. hyperplasia (increase in number) or hypertrophy (increase in size) [1,2]. This transient increase in β-cell proliferation to properly secrete sufficient amount of insulin is required to restore normal glucose level, yet defect in this process could have pathological consequences. Diabetes mellitus are multifactorial disorders characterized by an autoimmune β-cell destruction (type 1) or a combination of insulin resistance in peripheral tissues and β-cell failure (type 2). The resulting hyperglycemia is responsible for several long-term complications, such as retinopathy, neuropathy, kidney diseases or cardiovascular diseases. During type 2 diabetes, the chronic hyperglycemic state will enforce insulin secretion, and thus β-cell function, finally leading to a decrease in β-cell mass. Therefore, understanding how β-cells proliferate and function is of utmost importance and could have strong implications in the development of new therapeutic strategies for type 1 and 2 diabetes. Participation of cell cycle regulators in this process can be predicted, and has been demonstrated, as described below. However, the aim of this paper is to comment our recent data demonstrating that, in addition to their role in the control of cell mass, cell cycle regulators directly regulate β-cell functions [3].

## Discussion

### Cell cycle, β-cell and more

E2F transcription factors are often depicted as the ultimate effectors of the G1/S transition of the cell cycle. When bound to DNA, they exist either as free E2F/DP heterodimers, or associated in larger complexes containing members of the retinoblastoma family (pRB, p107, p130) and members of the cyclin/cdk protein families. Cyclin-dependent kinases (CDK) define a family of serine/threonine protein kinases that phosphorylate a number of substrates mainly implicated in cell cycle progression and transcription. Association of E2Fs with proteins of the pRB family facilitates active repression through recruitment of histone deacetylases [4] and lysine/arginine methyl-transferases [5]. Subsequent phosphorylation of the retinoblastoma protein by the cyclin/CDK complexes results in the release of the E2F transcription factors and activation of the transcription of genes required for progression through G1 into the S phase of the cell cycle. CDKs ultimately translate external signaling into transcriptional response, which is the final step of the regulatory cascade [6]. This makes the CDK-pRB-E2Fs potential candidates for the control of the crosstalk between proliferative stimuli and metabolic response. E2Fs functions are not restricted to the control of cell proliferation and it is now accepted that E2Fs participate in cellular processes beyond the cell cycle [7]. This has been extensively demonstrated through the characterization of *E2fs*-null mouse models [8] and the number of genes identified as E2F targets encoding factors involved in DNA replication, mitosis, DNA repair, apoptosis, differentiation, and development (reviewed in [9]). Of particular interest is the observation that E2Fs, pRB, and cdk4 could be involved in the regulation of a metabolic network, including the control of adipose tissue function [10,11].

Proliferation of β-cells is a key mechanism to maintain postnatal β-cell mass [12,13], and is the primary mechanism for β-cell regeneration [14-16]. It is now clear that pancreatic β-cells are able to replicate, albeit the origin of newly formed islets remains controversial. However, the mechanisms controlling β-cell replication unequivocally require cell cycle regulation. Gene inactivation studies of cell cycle regulators underscore the important role for these proteins in maintaining the β-cell population. Together with our previous observation that *E2f1 *-/- mice have decreased β-cell mass, due to decreased proliferation [17], other cell cycle regulators have been implicated in this process (for review, see [18]. Similar to *E2f1*-/-, *Cyclin D1*+/-; *Cyclin D2*-/- [19], *Cyclin D2 *-/- [15], and *Cdk4 *-/- [20] mice have decreased β-cell mass. Conversely, mice with gene inactivation of the cell cycle inhibitors p27 [21,22], p16^INK4A ^[23] have increased islet mass. Interestingly, genetic rescue of a mutant Cdk4 insensitive to INK4 inhibition (CDK4^R24C^) in a *Cdk4*-null background results in normal β-cell mass, suggesting a key role for CDK4 in controlling proliferation of β-cell [24].

### E2F1 in the control of β-cell function and insulin secretion

We previously evaluated the effects of E2F1 on glucose homeostasis. *E2f1 *-/- mice show an overall reduction in pancreatic size, as the result of impaired postnatal pancreatic growth [17]. E2F1 is not involved in embryonic β-cell development, since E2F1 is expressed at the final step of pancreas development, as demonstrated by in situ hybridizations. Because of the disproportionate small pancreas *E2f1 *-/- β-cells secrete insufficient amounts of insulin in response to a glucose load, resulting in glucose intolerance. However, despite this decreased insulin secretion, these animals were protected for the development of diabetes because of a dramatic increase in insulin sensitivity in other tissues, mainly adipose tissue [25]. In contrast, *E2f1/E2f2 *double KO mice are diabetic, showing exocrine pancreatic insufficiency, indicating that *E2fs *gene dosage and/or tissue specificity are critical for glucose homeostasis and pancreatic function [26,27]. These observations suggest however that E2F1 and E2F2 might cooperate to regulate β-cell mass and functions through cell-autonomous [3,17] and non-cell autonomous [26,28] effects. Surprisingly, we observed that E2F1 was highly expressed in non-proliferating adult pancreatic β-cells [17], suggesting that besides its impact on β-cell number, E2F1 could also play a role in pancreatic β-cell function and insulin secretion.

Pancreatic β-cell K_ATP _channels are composed by the heteromeric association of the sulfonylurea receptor 1 (SUR1) with the K^+ ^ATP channel inward rectifier (KIR6.2). These channels play a critical role in the regulation of glucose-induced insulin secretion by controlling membrane polarization [29,30]. The observation that *Kir6.2 *-/- mice display defective insulin secretion and increased insulin sensitivity is interesting [31], since it strongly resembles a particular phenotype of *E2f1*-/- mice [17]. Global gene expression analysis of *E2f1*+/+ and -/- pancreas revealed that the mRNA and protein levels of Kir6.2 were decreased. Most importantly, we demonstrated that *Kir6.2 *is a *bona fide *E2F1 target gene, as observed by chromatin immunoprecipitation performed on pancreas and isolated islet tissues. Most importantly, rescue of *Kir6.2 *expression in *E2f1 *-/- isolated islets restored glucose-stimulated insulin secretion, confirming that E2F1 exerts cell-autonomous effects on insulin secretion through its transcriptional control of *Kir6.2 *expression [3]. Furthermore, since *Kir6.2 *transcription is positively regulated by glucose, we studied the impact of glucose and glucose-stimulated insulin in the control of CDK4 activity and subsequent E2F1-dependent transcription. Our data demonstrated that glucose-stimulated insulin secretion triggers CDK4-dependent pRb phosphorylation in the β-cell, and subsequent transcriptional activation of the *Kir6.2 *gene by E2F1/DP-1 heterodimers. Finally, chemical invalidation of CDK4 activity, through the use of a specific inhibitor, resulted in glucose intolerance and impaired glucose-stimulated insulin secretion in mice, confirming a role for the CDK4-pRB-E2F1 pathway in insulin secretion process. Altogether, our data provide evidence that the CDK4-pRB-E2F1 pathway directly contributes to insulin secretion through the regulation of *Kir6.2 *expression in pancreatic β-cells both *in vitro *and *in vivo*. Our results reveal a novel feed-back control mechanism of the insulin response.

## Conclusion

We demonstrated that E2F1 transcriptional activity is controlled in pancreatic β-cells by insulin signaling secondary to glucose-induced insulin secretion [3]. How E2F1 can mediate both a proliferative (during post-natal growth or regeneration) or/and a metabolic response (during adulthood) in pancreatic β-cells in a coordinated manner is still not clear. Interestingly, insulin is an important β-cell proliferating factor, as evidenced by studies using β-cell specific inactivation of the insulin receptor (IR) in mice which resulted in a decrease in β-cell mass [32-34] or treatment of β-cell lines with IR siRNA which reduced proliferation rates of Min6 cells [35]. On the other hand, insulin has been shown to exert through an autocrine effect a feedback regulation on metabolic gene transcription, such as insulin itself (for review, [36]). Insulin signaling activation ultimately results in a specific metabolic response in β-cells. Since our recent findings indicate that the CDK4-pRB-E2F1 pathway is regulated by insulin, we can hypothesize that both the proliferative and metabolic effects of insulin on β-cells are mediated by the increase of CDK4 activity and subsequent E2F1 transcriptional activity. This would further suggest that both cell proliferation and metabolic responses are intimately linked, and regulated by the same upstream factors. In agreement with this hypothesis, caAKT^Tg ^transgenic mice deficient in *Cdk4 *display reduced β-cell proliferation, demonstrating that AKT induces b-cell proliferation in a CDK4-dependent manner [37]. Which is the pathway leading to cdk4-RB-E2F1 activation is largely unknown. One of the central elements that transduce signals from nutrient availability is the mTOR pathway.(Wullschleger et al., 2006). Strikingly, the metabolic phenotype of E2F1-/- mice is also reminiscent to the phenotype of mice carrying inactivating mutations in the mTOR substrate S6K1 (Pende et al., 2000; Ohanna et al., 2005; Aguilar et al., 2007; Um et al., 2004). Both E2F1-/- and S6K1-/- mice show impaired metabolism in pancreatic β-cells, adipose tissue, muscle, and likely in other tissues with metabolic functions, such as liver or brown adipose tissue. This strongly suggests a cross talk between the mTOR-S6K and cdk4-RB-E2F1 pathways. We speculate that the mTOR-S6K pathway controls metabolic processes, at least in part through regulation of the cdk4-pRB-E2F1 activity. The whole transcriptional program controlled by the CDK4-pRB-E2F1 pathway in endocrine pancreas remains, however largely unknown, and the signals and molecular mechanisms that underly the particular contribution of E2F1, CDK4 and pRB in metabolic *versus *proliferative response remains to be resolved.

We believe that the identification of new interacting pathways involved in the control of metabolism will open-up new perspectives for genetic studies in human population. The possible correlation between increased expression, or the presence of polymorphisms in any of the proteins studied in this work will help to identify a subset of subjects with increased risk to develop obesity-induced type II diabetes. Finally, we expect that this work will contribute to define new therapeutic strategies to combat type II diabetes.

## Competing interests

The authors declare that they have no competing interests.

## Authors' contributions

LF, EB, and JSA designed and wrote the manuscript.
